# A review on the effect of garlic on diabetes, BDNF, and VEGF as a potential treatment for diabetic retinopathy

**DOI:** 10.1186/s13020-023-00725-9

**Published:** 2023-02-17

**Authors:** Fatemeh Sanie-Jahromi, Zahra Zia, Mehrdad Afarid

**Affiliations:** Poostchi Ophthalmology Research Center, Department of Ophthalmology, School of Medicine, Shiraz University of Medical Sciences, Zand Boulevard, Poostchi Street, Shiraz, Iran

**Keywords:** Garlic, Diabetes, Diabetic retinopathy, BDNF, VEGF

## Abstract

**Background:**

Garlic is one of the favorite herbs in traditional medicine that has been reported to have many medicinal features. The aim of the current study is to review the latest documents on the effect of garlic on diabetes, VEGF, and BDNF and, finally, to review the existing studies on the effect of garlic on diabetic retinopathy.

**Main text:**

The therapeutic effect of garlic on diabetes has been investigated in various studies. Diabetes, especially in advanced stages, is associated with complications such as diabetic retinopathy, which is caused by the alteration in the expression of molecular factors involved in angiogenesis, neurodegeneration, and inflammation in the retina. There are different in-vitro and in-vivo reports on the effect of garlic on each of these processes. Considering the present concept, we extracted the most related English articles from Web of Science, PubMed, and Scopus English databases from 1980 to 2022. All in-vitro and animal studies, clinical trials, research studies, and review articles in this area were assessed and classified.

**Result and conclusion:**

According to previous studies, garlic has been confirmed to have beneficial antidiabetic, antiangiogenesis, and neuroprotective effects. Along with the available clinical evidence, it seems that garlic can be suggested as a complementary treatment option alongside common treatments for patients with diabetic retinopathy. However, more detailed clinical studies are needed in this field.

## Background

Garlic (Allium sativum) is a species of flowering plant belonging to the genus Allium [1]. This edible plant is widely used as a seasoning and flavoring, and its medicinal properties have been mentioned in various human and animal studies [2–4]. Garlic contains sulfur-based compounds (such as allicin, ajoene, diallyl polysulfides, vinyldithiins, diallyl sulfide (DAS), diallyl disulfide (DADS), and S-allylcysteine) as well as non-sulfur-active compounds that exert the garlic’s biological properties. Biochemically, the main ingredient in garlic is allicin (alyl 2-propentio sulfate), which has a heat-sensitive structure and degrades rapidly into sulfur compounds in response to high temperatures. Biologically active compounds formed by the degradation of allicin can reduce reactive oxygen species and, therefore, may play a significant role in the immune enhancement and treating disease [5]. Several therapeutic benefits of garlic include anti-infective [6], antioxidant [7], antimicrobial [8], and anti-cancer [9] effects that have been reported in previous studies. Recent studies have shown that garlic contains more than 200 chemicals including organosulfur compounds, volatile oils, enzymes, vitamins and a range of other biologically active molecules that lead to its medicinal effects. Garlic can prevent the development of cardiovascular diseases, regulate blood pressure, reduce blood sugar and cholesterol levels, and provide antibiotic, antifungal, and antiviral properties. Moreover, garlic has the ability to remove free radicals and can exert anti tumoral properties [10, 11]. A number of studies have investigated the pharmacokinetic profile of garlic compounds [12, 13]. In one of the recent studies, the permeability parameters of some garlic-derived organosulfur compounds and their membrane interaction have been investigated using the artificial immobilized membrane chromatography technique [12]. Based on this study, the permeation ability of garlic-derived organosulfurs is mainly dependent on the lipophilic/polar interactions of the chemicals [12] (Table 1).

**Table 1:** Molecular formula, chemical structure and some pharmacokinetic parameters of some garlic -derived organosulfurs^a^

OSCs	MF	ChS	RT (min)	HIA%	BBBP	pKa1 (acidic)	pKa2 (basic)	RB	TPSA	Tm	TE
S-allyl-l-cysteine	C6H11NO2S		3.93	81.972	No	2.53	9.14	5	88.62	7.758	6.222
Alliin	C6H11NO3S		3.71	73.041	No	1.84	8.45	5	99.60	8.386	11.192
Diallyl disulfide	C6H10S2		16.68	98.169	Yes	b	b	5	50.60	6.75	3.512
E-Ajoene	C9H14OS3		7.46	99.314	No	14.9	b	8	86.88	16.744	10.291
Z-Ajoene	C9H14OS3		7.41	99.314	No	14.9	b	8	86.88	13.945	10.421
Diallyl sulfide	C6H10S		9.71	100.000	Yes	b	b	4	25.30	6.067	3.223
Allicin	C6H10OS2		5.06	98.312	Yes	b	b	5	61.58	6.92	6.924
Diallyl Trisulfide	C6H10S3		25.6	98.996	Yes	b	b	6	75.90	8.334	3.549

*BBBP* blood–brain barrier permeation, *ChS* chemical structure, *HIA* human gastrointestinal absorption, *MF* molecular formula, *OSCs* organosulfur compound, *RB* Rotatable bonds, *RT* Retention time, *TE* topographic electronic descriptor, *Tm* total size index/weighted by mass, *TPSA* topological polar surface area

^a^For more information please see the work by Ramirez et al.[12]

^b^Non-ionizable compound

Garlic has been shown to play a protective role in cardiovascular disease [14]. In addition, garlic is advantageous for treating metabolic diseases such as diabetes [15]. Garlic can decrease the glucose level in mice and rats' serum [16]. The effect of garlic on blood glucose levels in diabetic mellitus (DM) has also been reported [17]. The focal point of this study is to review reports related to garlic's anti-diabetic, anti-angiogenesis, and neuroprotective impacts. Diabetic retinopathy (DR), the leading ocular problem in diabetic patients, is one of the world's most common causes of visual impairment [18]. The disease appears to be caused by retinal vasculopathy, retinal inflammation, and retinal neuropathy [19]. Hence, the new therapeutic strategies focus on controlling these processes at the molecular level. In this regard, the present study has focused on the effect of garlic on the expression of vascular endothelial growth factor (VEGF) and brain-derived neurotrophic factor (BDNF) at the cellular and molecular levels. This study is specific in terms of reviewing these two factors and the antidiabetic effect simultaneously. Besides, the clinical and experimental studies investigating the effect of garlic on the improvement of DR and retinal abnormality have also been reviewed in this study.

## Main text

In this study, after searching the Web of Science, PubMed, and Scopus English databases from 1980 to 2022, we extracted the most related English articles with these keywords: garlic, garlic extract, aged garlic extract, allium sativum, allyl compounds, DADS, diabetes, DM, type 2 diabetes, glucose parameters, anti-glycation, hypoglycaemic, insulin-resistance, DR, BDNF, neurogenesis, neuronal survival, VEGF, VEGF-related factor, and angiogenesis factor. All in-vitro and animal studies, clinical trials, research studies, and review articles, including the mentioned keywords, were assessed and classified.

Fresh garlic, aqueous extract of heat-treated garlic, garlic powder, aged garlic extract (AGE), and garlic oil (GO) were the most common forms of garlic under investigation in different studies. The enzyme alliinase, liable for converting from alliin (S-allyl cysteine sulfoxide) to allicin, is commonly inactivated by heat. As a result, the main component of the aqueous extract of heat-treated garlic is alliin. It has also been shown that the chemical composition of garlic powder is indistinguishable from fresh garlic [14]. AGE is delivered by placing the sliced garlic in 15–20% ethanol freshly soaked garlic at room temperature for a long time. The aging process diminishes the oil–solvent foul sulfur mixtures and upgrades the substance of water-dissolvable mixtures. The entire cycle should cause an impressive loss of allicin and expanded action of certain fresher mixtures, such as S-allylcysteine, S-allylmercaptocysteine, allixin, and selenium, which are steady, extremely bioavailable, and potentially antioxidant [20]. GO is generally prepared by the steam refining process. From more to less, steam-refined GO compounds include diallyl, allyl methyl, and dimethyl mono to hexasulfide [21].

**Fig. 1:**
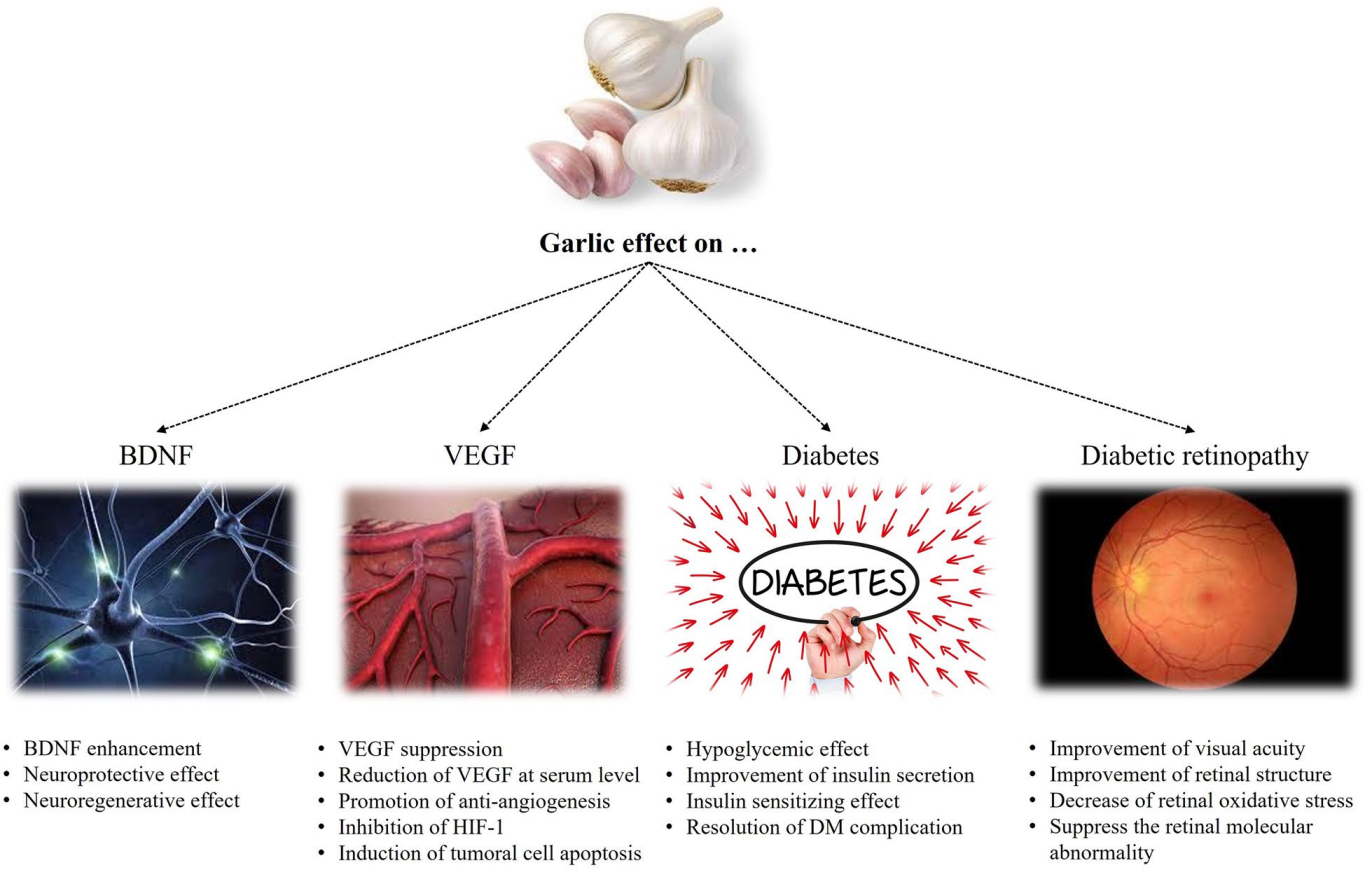
A schematic illustration of garlic effect on BDNF, VEGF, DM, and DR

The papers focusing on at least one of the forms of garlic compounds for managing diabetes, DR, and BDNF/VEGF level were included in the present study. Figure 1 represents a schematic illustration of garlic effect on BDNF, VEGF, DM, and DR. The collected data were classified as represented separately below (Table 2).

**Table 2:** A brief review of reports on the effect of garlic on diabetes, diabetic retinopathy, BDNF, and VEGF

Garlic (and its compounds)	The subject under study	Outcome	Refs.
***Garlic effect on diabetes***			
Aged garlic extract	STZ-induced diabetic rat	BMI (−), GLU (−), insulin (+), CL (−), TG (−), UP (−)	[32]
Garlic + met	T2D patients	Garlic: FBS (−), insulin (+), TC (−), risk of cardiovascular disease (−) Garlic + Met: more glycemic control and antihyperlipidemic activity	[15]
Garlic oil	STZ-induced diabetic rat	UP (−), GTT (+), insulin (+)	[36]
Chewing vs swallowed garlic	T2D patient (uncontrolled dyslipidemia	CL (−), TG (−), SBP (−), DBP (−), even with increasing fat intake; *Swallowed garlic had no significant effect on serum lipids	[22]
Raw crushed garlic	Metabolic syndrome patient	FBS and other metabolic syndrome elements (−)	[24]
Garlic extract	STZ-induced diabetic rat	GLU (−), insulin (+)	
Raw garlic extract	STZ-induced diabetic rat	GLU (−), CL (−), TG (−), UP (−)	[38]
VO (3mpa)2 complex	3T3-L1 preadipocytes STZ-induced diabetic mice	PI3K/Akt pathway (−), GLUT4 translocation to the plasma membrane (+), GLU (−)	[39]
Garlic oil /DATS	Diabetic rat	Insulin (+), GTT (+)	[16]
VO (alx)2 (Allixin isolated from dried garlic)	rat adipocytes T1D model mice	serum glucose (−), insulin resistance (+), GTT (+), *The plasma adiponectin level was not changed	[43]
Aqueous extract of raw garlic	alloxan-induced diabetic male rabbit	GLU (−), CL (−)	[45]
Aged garlic extract	STZ-induced diabetic rat	GLU (−), GHb (−), Renoprotective effect (+)	[38, 46]
Alliin (S-allyl cysteine sulfoxide)	DIO mice	Insulin sensitivity (+), CL (−), TG (−)	[47]
Garlic extract	T2D patient	GLU (−), CL (−)	[49]
Aged garlic extract	Immobilization stress in mice	renal cell hypertrophy (−), corticosterone (−), GLU (−)	[57]
Garlic	Metabolic syndromes in fructose fed rat	insulin sensitivity (+)	[64]
s-allyl cysteine-enriched black garlic juice	STZ-induced insulin-deficient mice	BMI (−), GLU (−), insulin (+)	[129]
Aged black garlic	db/db mice	insulin resistance (−), TC (−), TG (−), HDL (+)	[130]
***Garlic Effect on VEGF***			
Aged garlic extract	T2D patients at high cardiovascular risk	No significant change in endothelial cell function, vascular inflammation, oxidative stress or resistance to insulin	[23]
Aged garlic extract	Rat	Coronary arteries relaxation (+), myocardial contractility (−)	[77]
Alliin	Chick chorioallantoic membrane (CAM) model	Human endothelial cell proliferation (−), VEGF-induced angiogenesis (−) *The effect was boosted by the antioxidant vitamins C and E	[84]
Aged garlic solution	Chicken dorsum skin excisional lesion	Wound closure (+), re-epithelialization (+), dermal matrix regeneration (+), angiogenesis (+)	[88]
DAS	B16F-10 melanoma cell induced capillary formation in C57BL/6 mice	Tumor-directed capillary formation (−), IL-1 (−), IL-6 (−), TNF-α (−), GM-CSF (−)	[89]
Aged garlic extract	Colorectal carcinoma cells	Tumor growth (−), neovascularization (−), endothelial cell motility (−), endothelial cell proliferation (−), tube formation (−)	[91]
Garlic + lemon aqueous extraction	Mice with breast cancer	Angiogenesis (−), apoptosis (+)	[94]
DADS	Human prostate cancer cell line	PI3K/Akt (−), Ras/Raf (−), MMPs (−), pro-inflammatory/pro-angiogenic molecules (−), VEGF (−)	[96]
Garlic-derived extracellular vesicles	A549 human lung carcinoma cells	VEGF (−), programmed cell death (+), *Normal cells were unaffected by treatment	[98]
Garlic oil	Avian embryo chorioallantoic membranes	Microvessel (−)	[131]
Garlic powder	ductal breast carcinoma cells	VEGF (−), Angiogenesis (−)	[90]
Garlic stem extract	melanoma cells	Cell growth (−) cell migration (−)	[99]
***Garlic effect on BDNF***			
Aged garlic extract	Cerebral ischemia rat	8-OHdG (+), TNFα (+), COX-2 (+), The damage caused by neuronal injury (−)	[115]
DADS, DATS	Chronic constriction injury rat	Strong improvement in discomfort, H2S (+), BDNF (+), Nrf2 (+)	[116]
Allixin	Cultured neurons from fetal rat brain	Neurotrophic activity (+), nerve cells viability (+), division site per axon in hippocampal neurons (+)	[117]
Garlic essential oil	Mouse dentate gyrus	BDNF (+), AChE (−)	[118]
S-allyl-L-cysteine	Mouse dentate gyrus	Neuroblast proliferation (+), neuroblast differentiation (+)	[118]
Garlic essential oil	Rats utilizing the forced swimming test and unexpected chronic mild stress	The immobilization period (−), The sucrose preference index (+), 5-HT (+), DA (+), hippocampal BDNF (+), CREB (+), and protein kinase B (AKT) (+)	[119]
DADS	Hippocampal neural cell proliferation in the adult brain	Hippocampal neurogenesis (−), neurocognitive functions (−), BDNF (−), CREB (−), ERK (−)	[120]
DAS/DADS	Human malignant Neuroblastoma SH-SY5Y cells	Anti-apoptotic factors (−), calpain and intrinsic caspase cascade (+), hippocampal neurogenesis (−), neurocognitive functions (−), BDNF (−), CREB (−), ERK (−)	[121]
Garlic extract	Hippocampus cell in diabetic rat	hippocampus Na + /K + ATPase (+), Ca2 + ATPase (+)	[107]
DATS	diabetic rats heart tissue	NO_2_^−^(−), O_2_^−^(−), Bax, caspase-3 and -9 (−)	[113]
***Garlic effect on DR***			
Garlic	Pre-diabetes induced retinal abnormalities in high fructose fed rat model	Functional, structural, and molecular abnormalities of the retina (−)	[125]
Aqueous garlic extract	Diabetic Wistar rat	Retinal oxidative stress serum glucose (−), TGF-β2 (−), IL-1β (−)	[124, 126]
Garlic	Patients with diabetic macular edema	Visual acuity (+), CMT (−), IOP (−)	[128]

*AChE* Acetylcholinesterase, *BMI* body mass index, *CL* Cholesterol, *CREB* c-AMP response element-binding protein, *DADS* Diallyl disulfide, *DAS* diallyl sulfide, *DATS* Diallyl trisulfide, *DBP* diastolic blood pressure, *db/db mice* diabetic mice, *DIO* diet induced obese, *FBS* fasting blood sugar, *GHb* Glycosylated hemoglobin, *GLU* glucose, *GTT* glucose tolerance test, *HDL* high density lipoprotein cholesterol, *Met* metformin, *Nrf2* nuclear factor erythroid 2-related factor 2, *SBP* systolic blood pressure, *TC* total cholesterol, *TG* triglyceride, *T1D* type 1 diabetes, *T2D* type 2 diabetes, *UP* urinary protein

### Garlic and antidiabetic effects

The anti-diabetic effect of garlic has received much attention in the last two decades. [15, 22–30] The available documents have mainly investigated the effect of garlic on blood glucose control in healthy animals, animal models, and diabetic patients. [31] Diabetes was developed in animal models using chemical mixtures such as alloxan or streptozotocin (STZ). Alloxan or STZ do not cause actual type 1 DM due to differences in the immunologic mechanism or type 2 DM since it could not create actual insulin resistance. As an alternative, in such experimental investigations, DM was caused only by the injection of a toxic substance that affects the beta cells of the pancreas. The severity of this procedure depended on the dosing schedule and delivery technique. These types of studies had methodological limitations because these animals can live even without insulin injection, so they failed to create an entirely insulin-dependent state [16, 32–44].

Of 45 articles that were critically appraised for assessment of the anti-diabetic impact of garlic in this systematic review, 7 pieces of research were done in-vitro, 32 were done on the pharmacologically diabetic induced animals, 6 were clinical trials done on diabetic patients. The results of the antidiabetic impact of garlic are categorized in the 5-sub grouped as the discussion below.

#### Hypoglycemic effect of garlic

Lower blood glucose was meaningfully highlighted in the garlic-treated diabetic rats’ group compared to the control group. Raw garlic was shown to exert significant effects on hypoglycemia and hypocholesterolemia [45]. Eidi et al. reported lower serum glucose was obtained by oral administration of the garlic extract [33]. He described a persistent increase in blood glucose level in untreated control rats, while glucose level was significantly reduced in diabetic rats given 300 or 600 mg/kg of AGE. In contradiction with previous results, the blood glucose level in diabetic rats treated with a 100 mg/kg AGE dose was no longer substantially distinct from control ones. Glycosylated hemoglobin (GHb) ranges had been considerably reduced by administering the 300 and 600 mg/kg AGE doses. Treatment with the 100 mg/kg dose of AGE could no longer affect this parameter [38, 46]. This study found that the antidiabetic effect of AGE was dose-dependent.

Several investigations focused on the synergistic effects of garlic and anti-diabetic drugs like metformin on fasting blood glucose (FBS) levels in patients with type 2 diabetes [21, 26, 33]. Ashraf et al. described a statistically significant drop in FBS [15]. The effects of garlic extract and glibenclamide, a common anti-diabetic medication, were compared in diabetic rat models. The extract's anti-diabetic effect was more potent than glibenclamide's [33]. Therefore, garlic, combined with traditional anti-diabetic medication, has been demonstrated to enhance glucose control. FBS and other metabolic syndrome elements were considerably reduced when raw crushed garlic was used [24]. In alloxan-induced diabetic male rabbits, an aqueous extract of garlic -with very identical components to fresh garlic- provided a considerable drop in the elevated blood glucose level when tested to survey the physiological effect on serum glucose [45]. In addition to better glycaemic control, GO-treated diabetic rats revealed an increased in-vivo glycemic control and hypoglycemia sensitivity to insulin administration [29, 47]. The hypoglycemic action of GO is mediated by diallyl trisulfide (DATS), a fundamentally important component of garlic [16]. Scanga found that alliin is a substrate of LAT1, a cell membrane transporter that binds to alliin and plays a vital role in human metabolism, diabetes, and cancer [48]. Besides the investigation that shows garlic can help control diabetes, some studies suggest the contrary. According to Liu et al. study GO did not have antidiabetic effects immediately, but it took a few weeks for the result to appear [36].

Furthermore, DADS did not affect oral glucose tolerance [36]. Another study found that taking 900 mg of garlic orally, twice a day, did not reduce FBS or two-hour postprandial glucose levels in type 2 diabetes patients [49]. As more investigations indicate garlic has a favorable impact on blood sugar, the negative results of specific studies might be attributed to discrepancies in product preparation, consumption dosage, or duration.

#### Mechanisms for garlic as a hypoglycemic agent

The mixture of alliin, with usual antidiabetic drugs like glibenclamide and insulin, has been stated to have moderate effectiveness in regulating hyperglycemia [37]. Black solo garlic extract reduced the IL-1β, IL-6, and TNF-α level and increased IFN-γ in the STZ diabetic rats compared to glibenclamide. [50]. Recently Takim et al. showed that allium plays a role in the treatment of diabetes by enhancing the gene expression of *caspase 3* and *caspase 9* [51]. alliin modulates intestinal microbe composition, typically reducing Lachnospiraceae and augmented Ruminococcaceae in diet-induced obese (DIO) mice, and because of this effect, Zhui et al. concluded that garlic has a nutritional or therapeutic role in preventing diabetes [47]. In an animal study, Khare concluded that the use of allicin as an agonist of transient receptor potential ankyrin 1 (TRPA1), concurrently with a high-fat diet, could prevent GLP-1 dysregulation and glucose hemostasis [52]. It has also been hypothesized that the hypoglycemic effect of garlic is due to the presence and the impact of allylepropyl disulphide or diallyle disulphide and their effect on purine metabolism [16, 53, 54] Treating the rats with a combination of GO and DATS meaningfully increased the proportion of glucose to glycogen conversion. [16, 55]. In another study by Swanston, it was shown that the blood glucose regulating the activity of garlic extract was due to the presence of sulfur-containing combinations and flavonoids [56]. Kasuga. S also reported that garlic was effective in preventing the rise of corticosterone in response to adrenal hypertrophy, and because of that, it might avoid increasing blood glucose in diabetic mice [57].

#### Garlic and insulin secretagogues

Garlic is demonstrated to have insulin secretagogue properties, while the detailed mechanism is not apparent [15, 58]. Higher serum insulin levels resulted in diabetic rats that used the AGE (doses of 100, 300, or 600 mg/kg) but not in control rats [33, 38]. Moreover, insulin level was underlined to be increased in diabetic rats when given garlic in the form of oil [16] or garlic extract with a 200 mg/kg dose. Also, it may increase pancreas beta cell function by upregulation of the peroxisome proliferator-activated receptor-gamma coactivator-1α (PGC-1α) gene [59] and alter histopathological features of the pancreas cells [60]. S-allylcysteine sulfoxide appeared to have a direct stimulatory effect on the insulin secretion ability of the pancreas cells [16, 34]. It can stimulate the glucose transporter-4 and increase insulin secretion [61]. A recent study highlighted the role of protein tyrosine phosphatase 1B (PTP1B) negative consequences in the insulin signal pathways. Garlic and its inhibitory effect on PTP1B may help in DM type 2 treatment [62].

#### Insulin sensitizing effect of garlic

Moreover, garlic was found to deliver hypoglycemic effects by preventing insulin inactivation by the sulfhydryl group and increasing insulin-sensitizing [63]. Antioxidant components of aged black garlic, such as phenols and flavonoids, also reduce insulin resistance [28]. Padiya et al. described garlic’s potential to improve insulin sensitivity in fructose-fed rats [64]. Recently Parham et al. reported a garlic-containing herbal medicine that could elevate insulin secretion, sensitivity, and diminished insulin resistance [27]. About which active ingredient in garlic is responsible for this effect, Zhai et al. suggested that alliin may increase insulin sensitivity [47]. In addition, GO or DATS was shown to enhance oral glucose tolerance and increase insulin sensitivity [16]. As suggested by previous studies, the role of the other components of garlic named VO (alx) 2 (allixin isolated from dried garlic)- in the normalized hyperglycemia in mice- was also highlighted. This role was obtained by improving insulin resistance without any changes in the plasma adiponectin levels [39, 43, 65].

#### Effect of garlic on the DM complications

Longstanding high blood glucose associated with DM has a temporary or long-term effect on organs and tissues. Cardiovascular disease, stroke, renal illness, ocular problems, microvascular abnormalities such as neuropathy and nerve injury, foot problems, cutaneous problems, gastrointestinal problems, and various types of mental illness are all recognized as DM consequences. Micro-and macrovascular diseases linked with type 1 and type 2 diabetes have been widely discovered [65, 66]. Endothelial cell function and blood perfusion to the peripheral tissue measured by acetylcholine (Ach) provocation were improved after the AGE consumption in the cases of arterial arteriolosclerosis [67].

Furthermore, it directly acts on the arterial wall [68] and can elevate cystathionine-γ-lyase expression in the myocardium [69]. A regulation in the liver peroxisome *PGC-1α* and irisin-encoding gene expression, along with the other inflammatory cytokines, showed that the garlic oil could lighten diabetic liver injury [70]. The study on nephropathy, a common microvascular complication in DM, showed that the diabetic rats treated with garlic remarkably lowered urinary protein levels and renal CD36, podocalyxin, and NGAL in diabetic rats compared to the control ones [71, 72]. In another study, Thomson et al. showed that raw garlic could decrease kidney damage by reducing the urine protein levels in STZ-induced diabetes [38]. GO may also affect decreasing proteinuria at the end of the 16^th^ week of supplementation and improved oral glucose tolerance [36]. It seems that garlic can decrease the total cholesterol level by reducing LDL-C and thereby lower the risk of cardiovascular diseases [73]. Referring to the report of Ryu et al. aged black garlic could inhibit lipid oxidation by decreasing the free radicals [28]. Besides, it could effectively treat a patient with uncontrolled dyslipidemia (high cholesterol and triglyceride) and reduce blood pressure [25, 74]. Meanwhile, undamaged garlic (swallowed) did not have much effect on serum lipids [22]. Alongside raw garlic, AGE can also protect the cardiac and nervous systems and prevent clot formation [28]. Persaud H. reported that raw garlic consumption is associated with increased bleeding tendency [75]. In animal models' hearts, AGE improved ischemia–reperfusion by relaxing the outcome in coronary arteries [76, 77]. On the other hand, Atkin et al. developed a clinical trial on 26 subjects with type 2 diabetes who had high cardiovascular risk. The patients were treated with 1200 mg of AGE or placebo for 1 month, however, the results did not seem to confirm any improvement in the function of the endothelial cell, decreasing insulin resistance or oxidative stress [23].

#### Garlic and gestational DM (GDM)

Si et al. designed a randomized clinical trial to evaluate the effect of black garlic on GDM and concluded that insulin resistance was enhanced in the treatment group. The probiotic bacteria could improve the antioxidant effect of garlic in the GDM patient by converting the glucopyranoside in the fresh garlic to glucofuranoside, besides altering the intestinal’s normal flora [78].

### Garlic and VEGF

VEGF is a growth factor and a homodimeric glycoprotein. Its gene is located on chromosome 6p21.1. Endothelial, neuronal, and glial cell proliferation, migration, and cell survival can be affected using VEGF [79]. Hypoxia conditions and ischemia can stimulate VEGF expression. The previous investigations revealed that VEGF receptors are mainly located on endothelial cells; also, they can encourage ocular neovascularization with the help of receptors on the retinal cells [80, 81]. Hypoxia has been shown to stimulate VEGF expression. The primary genes involved in this process include stimulating hypoxia-inducible factor-1 (HIF-1), nitric oxide synthase (NOS), and VEGF genes. Hypoxia is a known etiological factor in various systemic and eye diseases [82]. Alliin, as part of the garlic component, displayed significant suppression of VEGF, resulting in anti-angiogenesis in the chick chorioallantoic membrane. Oral uses of the S-allyl cysteine can reduce the plasma level of VEGF and other pro-inflammatory cytokines such as interleukins (IL-1β, IL-4, IL-5, IL-10). [83]. This inhibitory effect was significantly boosted by the antioxidant vitamins C and E [84, 85]. Garlic can enhance cardiac angiogenesis by enhancing the expression level of myocardial miR-126 and miR-210. [86] Recent studies have shown that GO might reduce the number of microvessels in the avian embryo chorioallantoic membranes [87]. Besides, the histological analysis showed that AGE could improve the wound healing process in chicken dorsum skin excisional lesions, which was associated with dose-dependent neovascularization in AGE-treated injuries [88]. In rats, studies on the anti-angiogenic activity of DAS (a garlic component) showed the inhibition of tumor-directed capillary formation and reduction of pro-inflammatory cytokine production [89]. Hussein et al. reported that daily garlic powder could reduce intratumoral angiogenesis by decreasing the level of VEGF in the ductal breast carcinoma [90]. The anti-angiogenic effect was also reported in AGE-treated colorectal carcinoma [91] and glioblastoma [92], resulting in tumor growth inhibition of endothelial cell motility and proliferation. There is also a report on the same effect in hepatocellular carcinoma [93]. Furthermore, Talib Wamidhe found that combining garlic and lemon aqueous extraction reduced angiogenesis and caused apoptosis in mice with breast cancer [94]. It was shown that DADS could downregulate the PI3K/Akt and Ras/Raf signaling pathways in the human prostate cancer cell line through its inhibitory effect on HIF-1. This, subsequently, led to the downregulation of MMPs and several pro-inflammatory/pro-angiogenic molecules, namely VEGF expression. Hence it can be suggested that garlic possesses anti-invasive and anti-metastatic properties [95–97]. Using A549 lung carcinoma cells in humans as models and normal dermal fibroblast cells as controls, Özkan et al. demonstrated that garlic-derived tiny extracellular vesicles cause cancer cells to die by programmed cell death, although normal cells are unaffected by the treatment [98]. There is the study on the effect of garlic stem extract on the inhibition of the cell growth and migration in the melanoma cells [99]. As previously mentioned, various studies investigating the relationship between garlic and VEGF found that the main anti-VEGF effect of garlic is on apoptosis in malignant cell death and reduced angiogenesis in the in-vitro cell models.

### Garlic, BDNF, and neuroprotection

BDNF, a small size basic protein (with an isoelectric point of 9.6), is one of the most attractive members of the neurotrophin family [100]. BDNF plays a critical role in the growth and development of neurons. Its highest level of expression is in the human brain. BDNF has been shown to influence non-neuronal cells and their function in neuronal development. The liver, heart, and even lungs, express BDNF in smaller amounts [101]. Although it is commonly believed that newly produced hippocampal cells can be incorporated into neural networks during adolescence, neurogenesis usually occurs exclusively during embryonic development [102]. Hippocampal neurogenesis is critical for the generation of new synapses as well as the preservation of old ones. Numerous impulses that directly affect neurotransmitters, neurotrophic, and growth factors, such as BDNF, as well as a variety of environmental impulses, can control neurogenesis in adults [103, 104]. Herbal fortification can modulate hippocampal neurogenesis, both beneficially and adversely, even in adulthood [105, 106]. Semuyaba et al. noted the memory upgrading, increased hippocampus Ca^2+^ ATPase activity, and glutamine synthetase after ingesting a certain dosage of garlic in diabetic rats [107]. Numerous studies have been published to focus on the protective role of fresh garlic or AGE in neurodegenerative conditions such as Alzheimer's disease and cerebral ischemia [108–111]. Garlic might have a neuroprotective effect by enhancing tissue immunity against the oxidative stress caused by low-density lipoprotein [112], decreasing the free radicals such as NO_2_^−^ and O_2_^−^ [113], besides increasing mitochondrial function [114]. Colin. A. conducted a study on rats subjected to ischemia for 60 min plus 24 h of reperfusion. At the start of the reperfusion phase, the rats were supplemented with AGE (1.2 ml/kg weight). AGE reduced TNF levels as well as COX-2 protein amount and function. These findings imply that AGE's neuroprotective qualities are linked to both its anti-oxidant characteristics and ability to reduce TNF concentrations as well as COX-2 protein production and function. This study suggested that AGE could reduce the damage caused by neuronal injury [115]. In another study, DADS (25 and 50 mg/kg) and DATS (20 and 40 mg/kg) were given to chronic constriction injury (CCI) rats for 14 days. This study showed great discomfort after treating the rats with these garlic derivatives. Furthermore, H_2_S, BDNF, and nuclear factor erythroid 2-related factor 2 (Nrf2) amounts in the sciatic nerve and dorsal root ganglia were restored after administration of these organosulfur chemicals. DAS and DATS therapy of CCI-treated rats resulted in a considerable reduction in neuropathic pain. After nerve damage, the BDNF level in these areas decreased significantly. DAS and DATS therapy, on the other hand, resulted in a considerable recovery of the biochemical markers as well as a reduction in neuropathic pain [116]. Adding allixin (1–100 ng/ml) to the cell environment could markedly increase the viability of nerve cells obtained from different parts of the brain and expanded the number of division sites per axon in hippocampal neurons, as demonstrated by Moriguchi [117]. In another investigation, GO (10 ml/kg) was given orally to mice once a day, for three weeks. Subsequently, the analysis of the hippocampus homogenate confirmed a considerable elevation of BDNF concentration and a reduction in acetylcholinesterase (AChE) function [118]. The study of Huang Ju was the first to look into the antidepressant impacts of GO in rats utilizing the forced swimming test (FST) and unexpected chronic mild stress (UCMS). GO (25 and 50 mg/kg) effectively diminished the immobilization period in rats after 28 days of oral treatment. GO and DADS could also dramatically correct the reduction of sucrose preference index that was caused by five weeks of UCMS. With no hippocampal consequences, GO could substantially lower the frontal cortex recycling ratio of neurotransmitters such as serotonin and dopamine, raising their levels [119]. It has been shown that long-term administration of GO can augment hippocampal BDNF through monoamine neurotransmitter regulation and the BDNF-associated signaling cycle [119]. A study conducted on the dentate gyrus confirmed that S-allyl-L-cysteine, a constituent of the Allium class, can stimulate proliferation and differentiation in the neuroblast cells [118]. Despite the positive results of the effect of garlic on the increase of BDNF, some contradictory reports are also observed in this field. Recently the effect of sulfur components of garlic has been investigated on the proliferation of neural progenitor cells. This study showed that DADS remarkably downregulated the proliferation of these cells. Besides, the treatment of 10 mg/kg DADS decreased the hippocampus BDNF concentrations and subsequently reduced neurogenesis and lowered the function in the passive avoidance test. DADS could reduce the hippocampal level of BDNF, phosphorylated CREB signaling, and phosphorylated ERKs, all of that linked to neural stem proliferation and differentiation in the hippocampus. Furthermore, when compared to controls, DADS caused severe memory problems. Hence, DADS might have negative consequences on hippocampus neural cell proliferation and differentiation [120].

Garlic's effect on neurology system-related neoplasia has also been explored in keeping with its neuroprotective benefits. In neuroblastoma SHSY5Y cells in the human, Karmakar. et al. discovered that the GO components (DAS and DADS) stimulate the endogenous calpain-caspase pathway resulting in cell death. SH-SY5Y cells were treated with 50 and 100 M DAS or DADS for one day. This treatment led to the manifestation of cellular morphological hallmarks of apoptosis that was approved by Wright staining [121]. It seems that garlic can suppress neurological neoplasia.

Compared to non-diabetic adults, diabetic patients have lower levels of BDNF in their blood. Several studies have shown that the concentration of BDNF at the protein or mRNA level is reduced in the retina of diabetic rats [111, 122]. Considering the available evidence, it seems that garlic can enhance the neuroprotective effect in the management of diabetes in addition to improving the index related to glycemia. Although more studies in this field are needed, especially at the clinical level and in controlled conditions.

### Garlic and diabetic retinopathy

Notwithstanding numerous papers on the anti-diabetic effects of garlic, limited information has been reported on the impact of garlic on diabetic retinopathy [123]. The garlic components can reduce retinal oxidative stress and diabetic retinopathy by the effect of the Nuclear Factor kappa B (NF-ĸB) pathway and downregulation of its mRNA expression [124] . As reported by Kommula et al., the early supplementation of garlic at a rate of 3% in the daily diet of pre-diabetic Wistar rat models could prevent functional, structural, and molecular abnormalities of the retina. Pre-diabetic rats (n=9, in each of the control and case groups) in this study were modeled by use of high fructose diet for ten months. In this study, known molecular markers were used to track the abnormalities of a diabetic retinopathy model. VEGF and glial fibrillary acidic protein (GFAP) expression indicated the process of angiogenesis and glial activation in the diabetic retina, and increased expression of carboxymethyl lysine (CML-KLH), and 4-hydroxynanoenol (4-HNE) represented the association of glycation and retinal oxidative stress. This study demonstrated that garlic intake could significantly decrease the expression of VEGF, GFAP, CML-KLH, and 4-HNE in the retinal cells of the diabetic rat models compared to their control ones. This study provided valuable evidence on the potential of garlic intake to postpone retinal abnormalities manifestations [125]. Another study on streptozotocin-induced diabetic rats investigated the protective value of garlic intake on retinal abnormalities. Diabetic albino rats (n = 20, in each control and case group) were supplemented with raw garlic (0.4 g/100 gram of body weight) for seven weeks. Then after retinal samples were examined for tracking of any histopathological and ultrastructural alteration. It was found that the treated samples had morphological and structural improvements [123]. The anti-oxidative and anti-inflammatory effect of aqueous garlic extract on the retinal tissues of rats affected with DM has also been recently reported. Male Wistar rats (n=6 in each of the control and case groups) were modeled for diabetes by applying streptozotocin and nicotinamide. The modeled rats were supplemented with aqueous garlic extract (200 mg/100 gram of body weight /day) for 5 weeks and further analyses were performed on the homogenate’s lysis of retinal samples. This study supported the hypnotized of the reducing effects of garlic on the standard parameters associated with retinal oxidative stress. Besides, the expression level of TGF-β2 and IL-1β was indicated to be significantly decreased in the garlic-supplemented group [126, 127]. Recently, in a randomized clinical trial on 117 eyes of diabetic patients, Afarid et al. have shown that garlic intake could remarkably improve visual acuity, decrease central macular thickness, and reduce intraocular pressure. This study suggested that garlic can be a complementary treatment for diabetic macular edema [128].

## Conclusion

In conclusion, while the results of a few studies share several similarities in the impact of garlic on each of the antidiabetic, antiangiogenesis, and the neuroprotective effects were opposing and conflicting, the vast majority of many other research findings with a beneficial influence cannot be overlooked, and the positive impact of garlic can be supported in overall. This review was unique in that it looked at antidiabetic, neuroprotective, and antiangiogenic effects of garlic simultaneously. Also, the latest studies on garlic's therapeutic effect on DR were included in this study. According to the available evidence, it seems that garlic can be prescribed as a complementary treatment for DR patients. More research is needed to discover particular chemicals in garlic or garlic components accountable for most of its biological effects, such as antidiabetic, neuroprotective, and antiangiogenic actions.

## Data Availability

The data used to support the findings of this study are available from the corresponding author upon request.

## Abbreviations

4-HNE: 4-Hydroxynanoenol
AChE: Acetylcholinesterase
AGE: Aged garlic extract
BDNF: Brain-derived neurotrophic factor
CML-KLH: Carboxymethyl lysine
DADS: Diallyl disulfide
DATS: Diallyl trisulfide
DAS: Diallyl sulfide
DIO: Diet-induced obese
DM: Diabetic mellitus
DR: Diabetic retinopathy
FBS: Fasting blood glucose
FST: Forced swimming test
GDM: Gestational DM
GFAP: Glial fibrillary acidic protein
GHb: Glycosylated hemoglobin
GO: Garlic oil
HIF-1: Hypoxia-inducible factor-1
IL: Interleukin
NF-ĸB: Nuclear Factor kappa B
PGC-1α: Peroxisome proliferator-activated receptor-gamma coactivator-1α gene
PTP1B: Protein tyrosine phosphatase 1B
STZ: Streptozotocin
TRPA1: Transient receptor potential ankyrin 1
UCMS: Unexpected chronic mild stress
VEGF: Vascular endothelial growth factor
